# A case series of successfully managing exomphalos major with awake graduated compression dressing and early enteral feeding

**DOI:** 10.1007/s11845-024-03630-8

**Published:** 2024-02-20

**Authors:** Hussam Widatella, Sami Abd Elwahab, Zakya Penny, Sri Thambipillai Paran

**Affiliations:** Department of General Paediatric Surgery, Children’s Health Ireland at Crumlin, Dublin 12, Ireland

**Keywords:** Exomphalos major, Non-surgical management, Paediatric surgery

## Abstract

**Introduction:**

Exomphalos anomaly is defined as the herniation of abdominal viscera into the base of the umbilical cord, with only a membranous sac covering these contents. It has an incidence of approximately 1 in 4000–6000 births. Management of exomphalos major (EM) remains controversial and limited, with very few studies to guide decision-making.

**Method:**

This is a case series of four neonates with EM treated at a tertiary paediatric referral centre between 2018 and 2021 with a gradual compression dressing technique.

**Results:**

Four neonates were diagnosed with EM. The average gestational age was 38 + 5 (range 38 + 2 – 39 + 2), and the average birth weight was 3.1 kg (range 2.56 – 3.49 kg). The defect size ranged between 5 and 7 cm. All patients were commenced on gradual compression dressing between days 1 and 3 of life. Dressings were applied at the bedside in the general neonatal ward. The average time taken to reach full feeds was 1 week; only one patient required parenteral nutrition. Three underwent surgical repair at two and 16 weeks of age; one had delayed repair at the age of 1 year because of the COVID-19 pandemic. None required patch repair. None required prolonged ventilation after repair.

**Conclusion:**

This case series describes a successful compression dressing technique that reduces sac content without the need for general anaesthetic or respiratory compromise, whereby simultaneous enteral feeding is tolerated.

## Introduction

During embryonic development, the abdominal wall is formed by the infolding of the cranial, caudal and two lateral embryonic folds. In the fourth week of development, the four folds meet to form the umbilical ring. During the sixth week of gestation, rapid growth and herniation of the midgut into the cord occurs with subsequent return to the abdominal cavity at week ten [1]. Failure of this folding process can result in the abdominal viscera remaining outside of the abdominal cavity [2]. Ladd and Gross define exomphalos as the herniation of abdominal viscera into the base of the umbilical cord with the covering of these contents by a membranous sac [3]. The sac is composed of layers of the umbilical cord from superficial to deep: amnion, Wharton’s jelly, and peritoneum [2, 3].

Exomphalos is classified into minor, major, or ruptured [4]. The four criteria that define Exomphalos Major include an abdominal wall defect of 5 cm or more, viscero-abdominal disproportion, herniation of the liver, and a defect that cannot be closed primarily [3]. Exomphalos defects occur in approximately 1:4000 to 6000 live births [2]. This incidence is believed to have declined in recent years as a result of improved antenatal diagnosis and pregnancy termination of those with associated complex genetic disorders and malformations [4, 5].

## Method

Data were collected both retrospectively and prospectively, adhering strictly to the four criteria defining EM. Patient demographics and clinical photography were collected after obtaining informed parental consent. Cases with significant cardiopulmonary anomalies were excluded. Jamovi (version 2.3.26) software was used for analysis. Descriptive statistics were done, including mean, median, range and standard deviation were applicable.

## Management

**Fig. 1:**
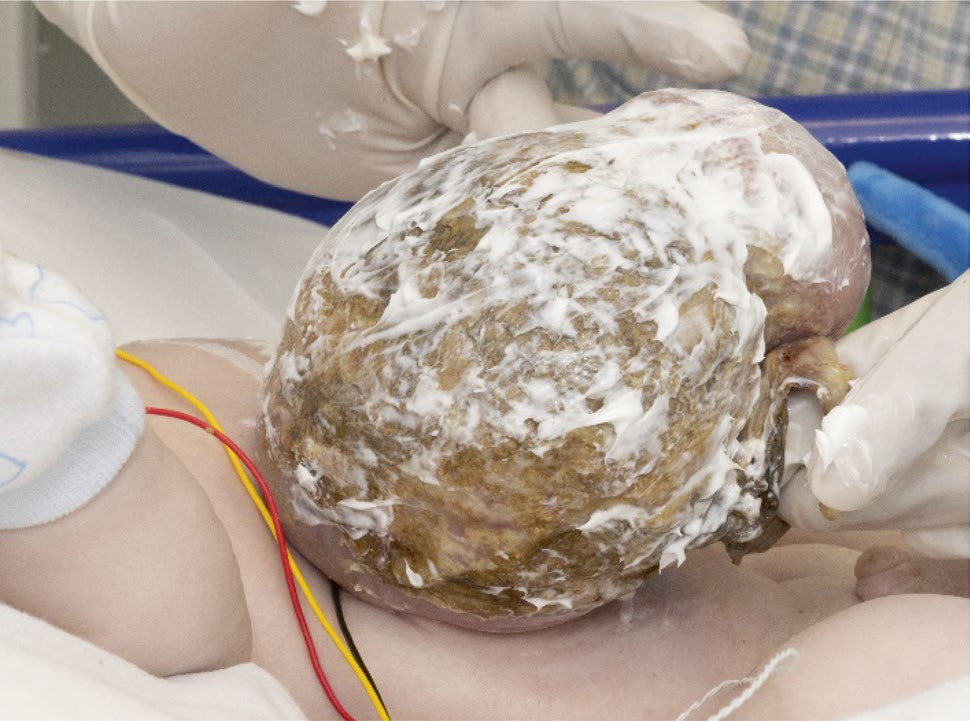
The first layer showing application of Silver Sulfadiazine (FLAMAZINE^™^)

**Fig. 2:**
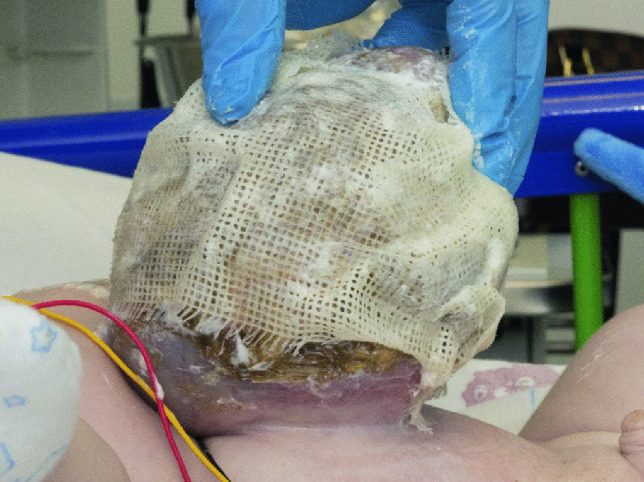
The second layer is a non-adhesive dressing (Mepitel^™^)

**Fig. 3:**
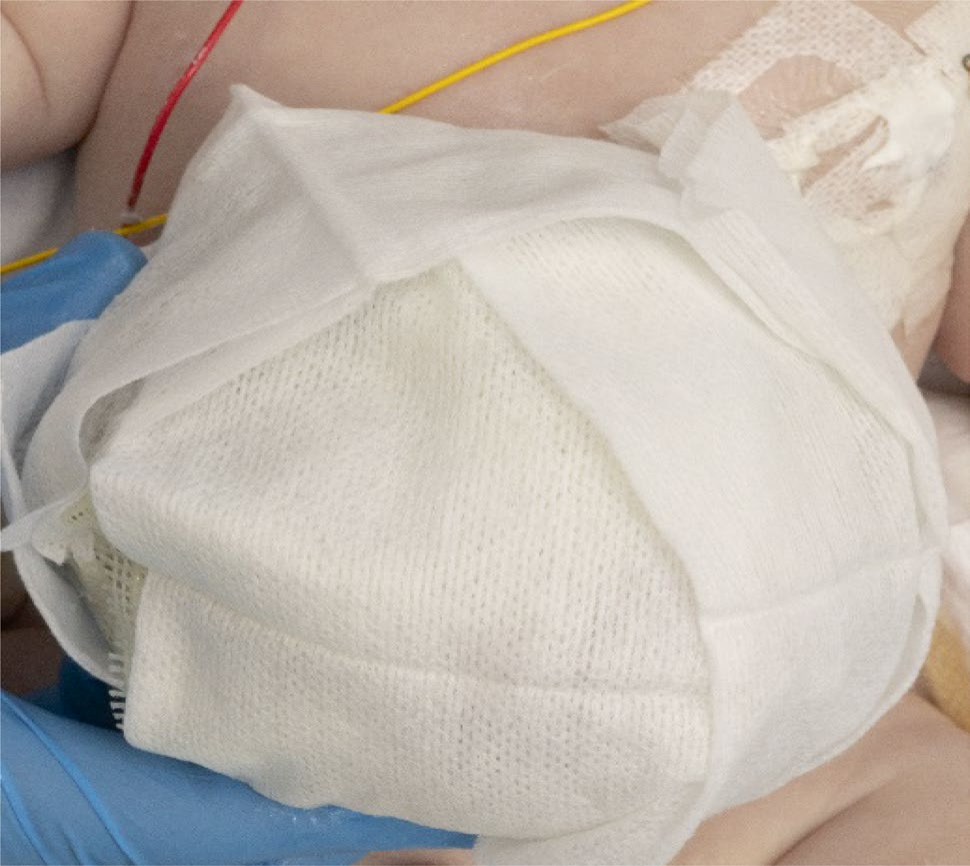
The third layer is clean dry gauze (Sofsorb^™^ Sterile Non-Woven Swabs)

**Fig. 4:**
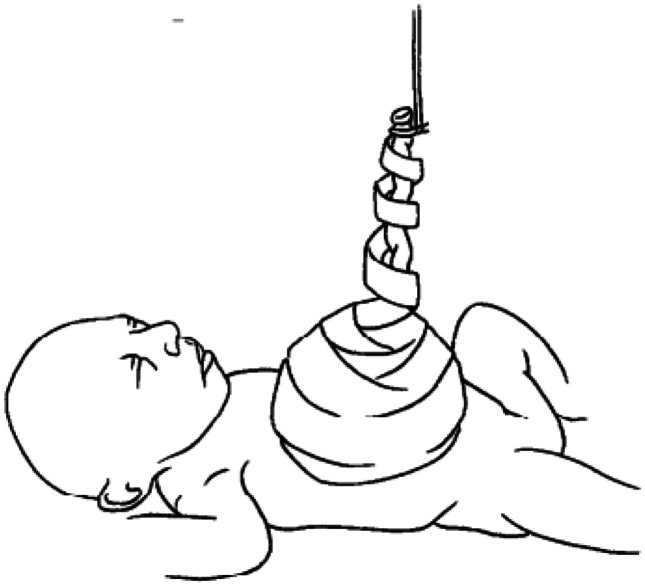
Umbilical cord suspension

A layered dressing is applied to the exomphalos sac — first the sac is painted with silver sulfadiazine (FLAMAZINE^™^) (Fig. 1); then a non-adhesive dressing (Mepitel^™^) is applied (Fig. 2). Dry gauze (Sofsorb^™^ Sterile Non-Woven Swabs) is applied (Fig. 3), and finally, this is covered by an elastic bandage (Mollelast^®^ 12 cm × 4 m). Initially, the umbilical cord is suspended using tape to hold the sac upright — (Fig. 4). This allows gravity to aid the gradual reduction of sac content while preventing the sac from leaning to one side, which might cause stretch and pressure on the other side and risk of rupture and/or strangulation of contents.

**Fig. 5:**
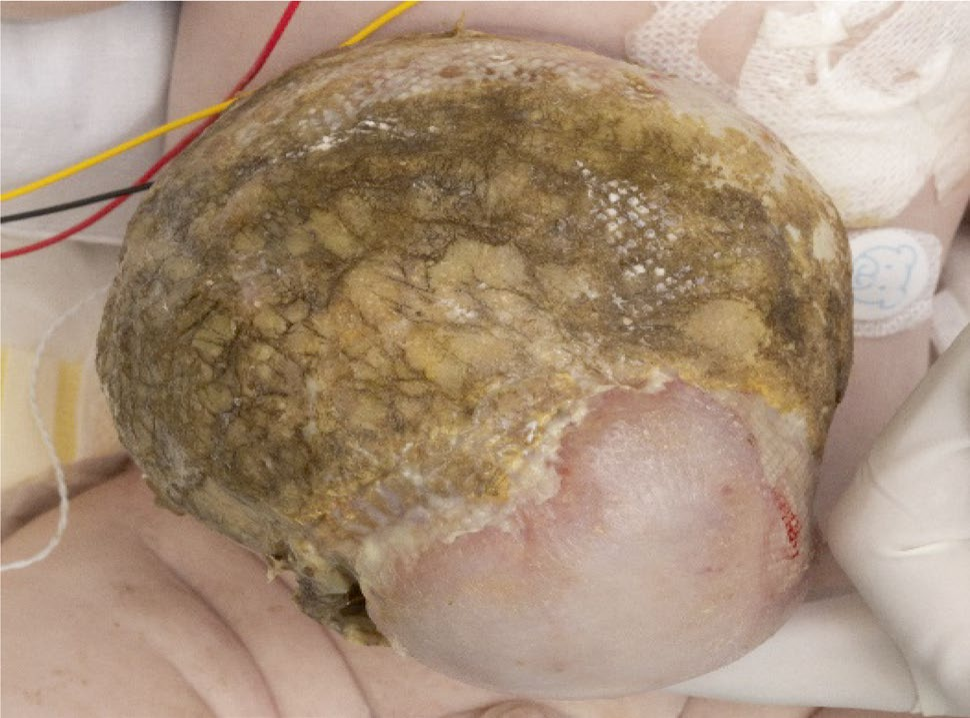
EM sac showing eschar formation 4 weeks after dressing application

**Fig. 6:**
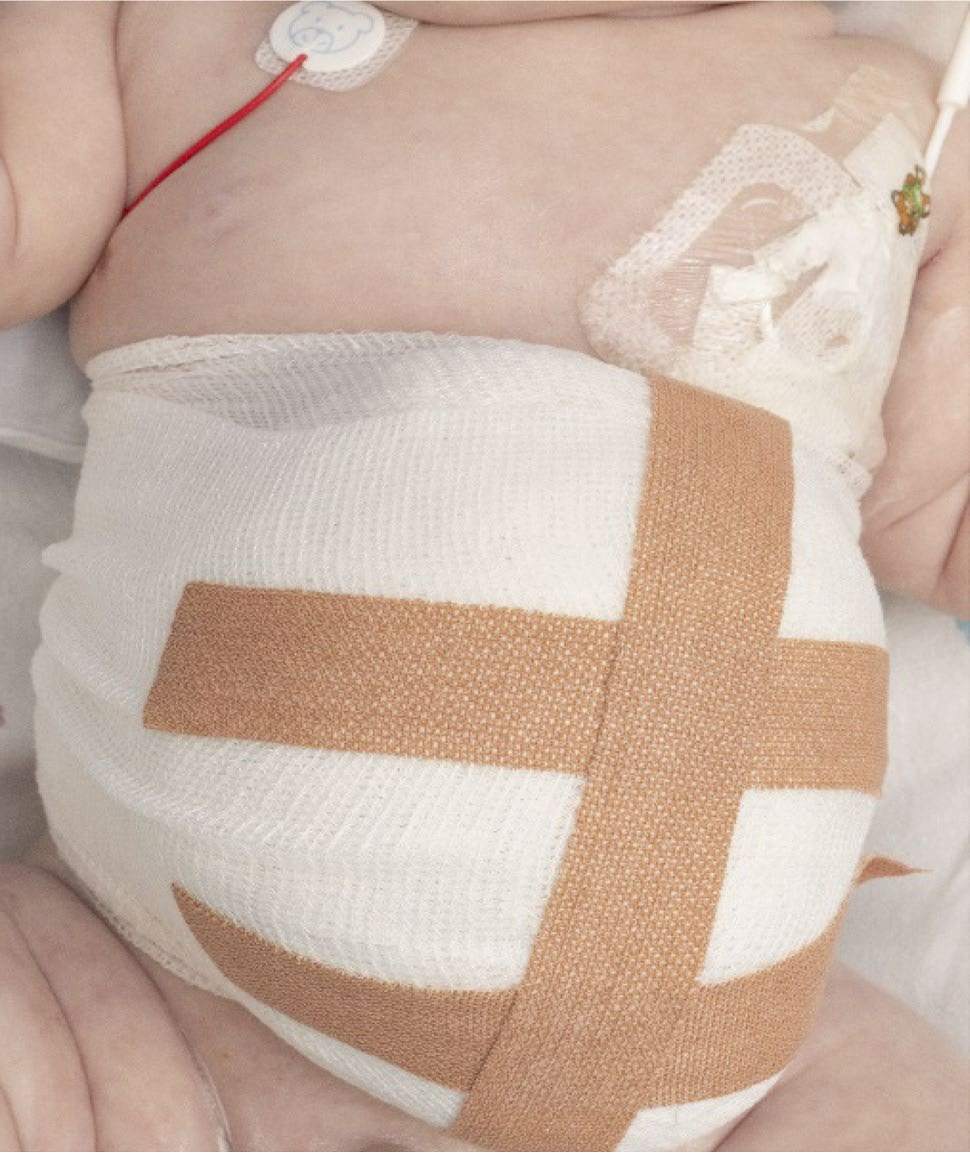
(Mollelast^®^ 12 cm × 4 m) is wrapped around the EM sac and around the lumbar region

Once the sac is dry and covered by an eschar (Fig. 5), umbilical cord suspension is stopped, and the elastic bandage is applied for the first time over the sac and around the lumbar region (Fig. 6). To create a pressure gradient, the elastic bandages are applied tighter at the top part of the sac, aiding in the gradual reduction of sac content into the abdominal cavity and reduction of the sac size. The dressing is changed at regular intervals, allowing sac examination and assessment of suitability for closure.

After application of the dressing, the patient is monitored for signs of irritability, respiratory distress, vomiting and all other features of compartment syndrome. Closure is considered when the contents can be manually compressed into the abdominal cavity without tension at the bedside.

## Case presentation

All patients in this series had normal antenatal karyotyping, microarray, and normal Apgar score at birth, and they all underwent a full neonatal physical assessment, echocardiography, renal and cranial ultrasound (US). Anomalies other than EM are described on a case-by-case basis below.

### Case one

A male of 39 + 2 weeks gestational age with a confirmed antenatal diagnosis of EM at 23 weeks gestation was born via elective caesarean section. His mother was diagnosed with maternity-related hypothyroidism and was on levothyroxine. His birth weight was 3.22 kg. He was transferred to our tertiary paediatric referral centre at 5 h of life.

In the immediate post-natal period, he was kept nil per orum and commenced intravenous fluids. On arrival, he was self-ventilating, maintaining his oxygen saturation above 96% on room air. Both cardiac and respiratory examinations were unremarkable, and no dysmorphism was noted. He had normal male external genitalia, both testes palpable within the scrotum and a normally sited anus. A routine echocardiogram showed a small apical ventricular septal defect (VSD). His cranial and renal US were normal.

The EM contained bowel loops and the liver. The sac was intact. The diameter of his abdominal wall defect was measured at 5 cm, and the liver shadow was visible at the superior aspect of the sac. Gradual compression dressing was applied on day 1 of life. The patient was commenced on small-volume oral feeds on day 1 of life and gradually progressed to full feeds by day 3 of life. After 5 days, the sac was noticed to be dry and covered by eschar, so the umbilical suspension was stopped, and the gauze bandage was applied to the sac and lumbar region. He underwent surgical closure of EM at the end of the second week of life.

**Fig. 7:**
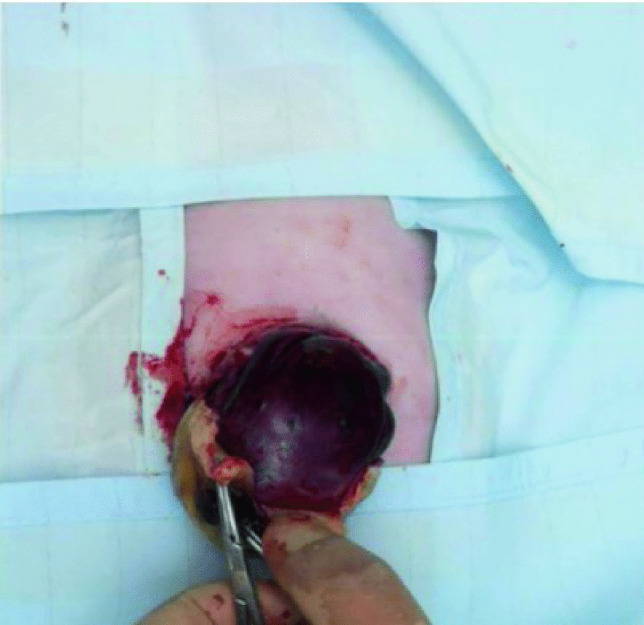
The cranial surface of the sac was adherent to the antero-superior surface of the liver

Intraoperatively, the EM sac was noted to be dry, intact, and containing liver only. The upper surface of the sac was adherent to the antero-superior surface of the liver (Fig. 7). The sac was excised except for the part that was adherent to the liver. The abdominal wall defect was incised cranially, and caudally to allow reduction of the liver into the peritoneal cavity. The rectus sheath was defined with minimal dissection and closed with a 2–0 Ethibond continuous suture. An umbilicoplasty was performed using 5–0 Caprosyn sutures.

Postoperatively, the patient was kept fasting with an NGT in place for decompression. He was commenced on intravenous antibiotics and nurse-controlled analgesia (NCA). On the first postoperative day, he developed tachycardia, increased work of breathing and abdominal distension. He was transferred to the high dependency unit (HDU) and commenced on supplemental oxygen via nasal prongs. Pain management was optimized, and his septic work-up and chest x-ray were unremarkable.

**Fig. 8:**
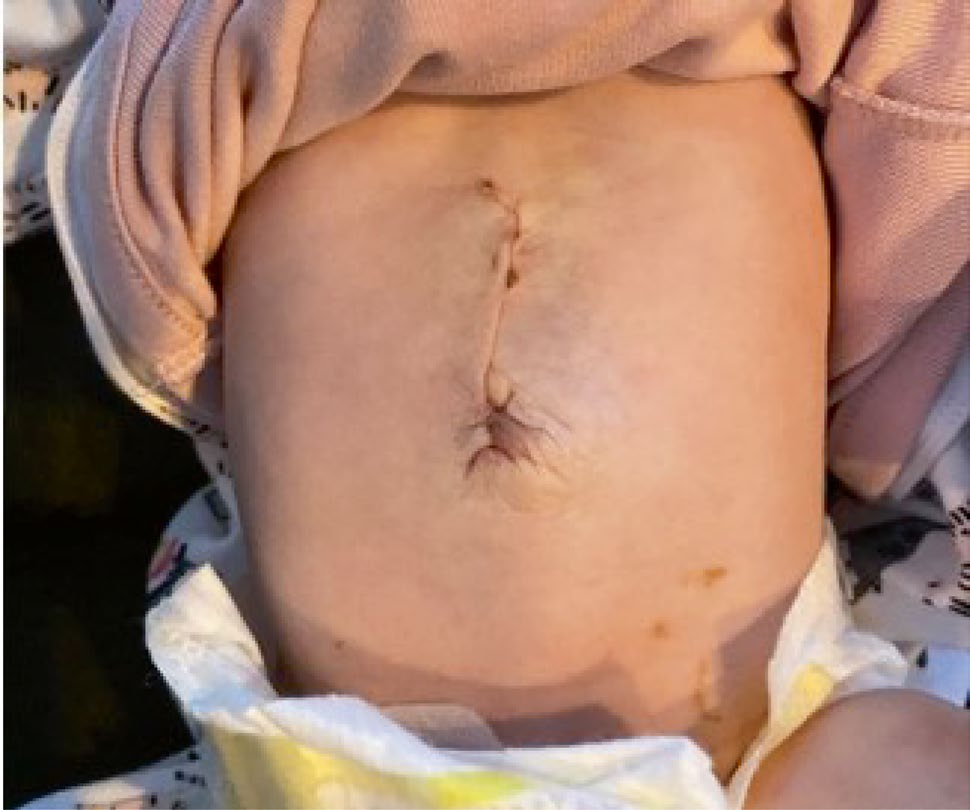
Follow-up in 3 months shows an int act EM repair

On the third postoperative day, oral feeding was restarted, and he was discharged from the HDU to the general ward. He tolerated full oral feeds and was discharged home on his eighth postoperative day. Three weeks following discharge, he presented to the emergency department with a reducible left inguinal hernia, and he underwent a left inguinal herniotomy the following day. He was discharged home less than 24 h postoperatively. On routine follow-up 3 months later, the EM repair was intact. (Fig. 8).

### Case two

Our second case is a female baby with a gestational age of 38 + 5. She had an antenatal diagnosis of EM confirmed at 35 weeks, with a small inlet VSD. She was born by elective caesarean section to a healthy mother. Her birth weight was 3.49 kg. She was transferred from the maternity hospital to our tertiary paediatric referral centre at 3 h of life.

On arrival, she was self-ventilating. Her EM defect was measured at 6 cm and contained liver and bowel. Additionally, the patient was noted to have dysplastic left middle and index fingers. She had a patent and normally sited anus, and she passed meconium on day 1 of life. An echocardiogram showed a 4 mm atrial septal defect (ASD) and a small VSD with left to right shunt. US of the urinary tract and cranial contents were normal.

Gradual compression dressing was applied on the first day of life. Simultaneous parenteral nutrition and trophic oral feeding were commenced on day 1 of life and gradually increased to full oral feeding on day 8 of life with cessation of parenteral nutrition. Given the size of the sac and its contents, the patient was discharged home with planned delayed repair. The dressing was changed every 3 days at the surgical day unit. At 4 months of age, she underwent surgical repair.

An elliptical 6 cm incision was made around the neo-epithelialized sac, taking care to remain extraperitoneal. On opening the peritoneum, blunt finger dissection was used to detach the colon from the sac. The fascial edges were defined with minimal dissection from the skin edges to preserve blood supply. The viscera were inspected to rule out malrotation or obstruction, and no bands were identified. The fascial defect was closed with 2–0 Ethibond. The subcutaneous layer was closed using 3–0 undyed Polysorb and the skin with 5–0 Caprosyn.

Oral intake was re-introduced postoperatively on the day. The patient was discharged home on day 2 postoperatively. A 3-cm diameter weakness was felt along the suture line on her annual review. This was treated expectantly; on review 2 years postoperatively, it completely resolved, with no weakness or hernia noted.

### Case three

A female baby of 38 + 2 gestational age was transferred from a maternity hospital at 3 h of life with EM. She was born to a healthy mother. Antenatal ultrasound confirmed EM at 24 weeks. The patient was born via elective lower segment caesarean section. Her birth weight was 3 kg.

The exomphalos sac was intact. The defect size measured 7 cm, and the sac contained the liver only. She had a patent normal-site anus and normal external genitalia. A routine echocardiogram showed a structurally normal heart; cranial and renal US were normal.

The patient was commenced on trophic feeds with maternal expressed breast milk (EBM) on day 1 of life and gradually increased in volume; full volume was reached on day 8. Gradual compression dressing was commenced on day 1 of life. The patient had a transient episode of desaturation on day 5, but it resolved spontaneously. The parents were trained in managing and changing the compression dressing with the help of a multidisciplinary team, including occupational therapists, physiotherapists, and clinical nurse specialists.

The patient was discharged home after 4 weeks in the hospital. Surgery was performed 3 months later. The defect was 6 × 6 cm in size and contained liver alone. A midline incision was made, the redundant skin was excised, the bowel was inspected, and the sac contents were reduced. The fascial edges were dissected, and the defect was closed using a continuous 2–0 Ethibond suture with mild tension. She was kept in a general ward postoperatively and commenced on feeds day 2 postoperatively. She was discharged home on day 3 postoperatively.

The patient was reviewed in the clinic 4 weeks later. She recovered well following her surgery and had an intact repair on examination.

### Case four

A female baby, with gestational age 38 + 2 weeks, had a confirmed antenatal diagnosis of EM. Born via emergency LSCS to a primigravida mother. Her birth weight was 2.56 kg. The patient was transferred to the tertiary paediatric centre at 24 h of life.

On assessment, she was on room air. Her cardiac and respiratory exams were unremarkable, and no dysmorphic features were noted. The EM defect was measured at 6 cm; the sac was intact and contained bowel and liver. The echocardiogram showed a patent ductus arteriosus and a small VSD. Both cranial and renal US were normal.

Gradual compression dressing was commenced on day 2 of life. EBM feeding was commenced via NGT and advanced gradually to reach full volume feeds on day 9 of life. The parents were trained on how to apply the compression dressing, and the patient was discharged home at 6 weeks of age. Surgical repair was planned at 11 weeks of age; however, it was delayed because she developed a lower respiratory tract infection at the time.

In an outpatient clinic follow-up at 4 months of age, she was found to have bilateral inguinal hernias, which were repaired a week later. Delayed EM closure was planned at 6 months of age. Unfortunately, due to the COVID-19 pandemic, this was further delayed until eventually performed when she was 1 year old.

Intraoperatively, an elliptical incision was made around the redundant abdominal skin. She was noted to have malrotation, so Ladd’s procedure was performed. Skin flaps were raised, and the fascial defect was closed with Ethicon 2–0; an umbilicoplasty was undertaken.

Postoperatively, she was commenced on oral feeds on the second postoperative day and was discharged home on day 3. On routine follow-up 1 year later, the repair was intact, and there was no evidence of a hernia. The patient was referred to plastic surgery for further umbilicoplasty.

## Discussion

The management of EM remains controversial and limited as there are few studies published to guide decision-making. Approximately 37% to 67% of EM cases are associated with additional congenital anomalies [6–8], such as chromosomal anomalies — mainly Trisomy 13, 18, and Beckwith-Wiedemann syndrome [8]. Pulmonary hypoplasia can be seen in up to 37% of EM patients [3, 9], and many also have associated cardiac defects [5, 6]. Despite improved management, the mortality rate remains as high as 17% to 41%, dependent mainly on the presence of associated anomalies [6, 7].

The size of the abdominal wall defect and its contents, the viscero-abdominal disproportion, and associated anomalies, especially pulmonary hypoplasia, and cardiac anomalies, determine the outcomes of the surgical treatment [10]. In EM patients with limited abdominal capacity, early closure of the abdominal wall defect can result in a sudden increase in intra-abdominal pressure, leading to splinting of the diaphragm and thorax, causing respiratory failure, reduced cardiac output, bowel ischemia and anuria [5].

Two distinct strategies of EM management have been described. The first strategy is staged surgical closure, involving multiple operations prior to final fascial closure and abdominal wall repair. Several techniques of surgical repair were described, including the placement of a sutured Silastic Silo, development of skin flaps, component separation procedure, mesh repair, vacuum-assisted closure, and intra-abdominal/subcutaneous tissue expanders [4, 5]. The disadvantage of these techniques is that they require multiple operations under general anaesthetic, with an increased risk of ventilator dependence and sepsis [5].

The second and more commonly utilised strategy describes a period of non-operative management with escharotic material followed by delayed surgical closure. In 1957, Grob et al. first introduced the concept of coating the sac, initially using 2% Mercurochrome [14]. A variety of topical medications have been employed in dressing the sac, most recently Sulfadiazine and Bovidone-Iodine. All medications are utilised to induce eschar formation and subsequent epithelialisation. This is followed by interval repair of the ventral hernia. Epithelialization of the sac usually takes between 6 and 15 weeks [11]. Delayed closure can be achieved after 10 to 14 months [12]. Several reports have confirmed the safety of this approach, with lower mortality rates and a shorter time to full feeds achieved [2, 3, 13].

The disadvantage of this delayed closure is that it eventually requires an operation to close the large ventral hernia, which is challenging. Systemic toxicity of various agents used to promote epithelialization has been reported; betadine is known to cause suppression of thyroid hormones [8]. Protracted treatment with this method is associated with high infection rates [3, 14], not to mention the economic and social burden on parents and healthcare systems.

This study describes a novel perioperative technique in a tertiary paediatric surgical referral centre. These cases demonstrate the successful implementation of an identical technique across defects of various sizes that can be used safely and easily. Historically Barlow et al. describe a technique of application of a silo dressing on a small case series of three patients with giant exomphalos (defects measuring 10 cm or greater by their definition), two of whom suffered serious respiratory compromise, all of whom were managed with nasogastric tubes (NGT) and serial aspiration. This case series includes four patients with EM (defects measuring 5–7 cm), none of whom suffered severe respiratory compromise, none requiring long-term NGT or aspiration [15]. Prior to Barlow et al., the use of prosthetic silo is described, with associated complications such as intestinal fistula formation, silo-associated sepsis, and component separation. Other techniques, such as coating the exomphalos sac to induce epithelialisation with mercurochrome and alcohol, were associated with failure of the abdominal cavity to enlarge [16]. The technique we described in this series involves suspension of the silo, thereby decreasing the pressure on the abdominal cavity and reducing the risk of respiratory compromise. Graduated compression dressing of the exomphalos sac negates the need for NG aspiration, with patients reaching full feeds quickly.

This series provided long-term follow-up for these patients. One of the limitations of this series is the lack of testing the serum silver levels. This was mitigated by the close clinical follow-up for signs of toxicity while being used. None of the cases described developed any symptoms or signs of toxicity. While not entirely new, the technique described in this report allows for the early introduction of enteral feeding, the possibility of early discharge and the active involvement of parents in the management. A larger prospective cohort of patients treated with this approach is probably needed to validate our results.

## Conclusion

This case series shows that EM patients can be treated safely by gradual compression dressing with a period of non-operative management followed by early surgical repair. The dressing can be changed on the ward or at home with adequate parent training and education, minimising the length of hospital stay. All patients described in this series were commenced on enteral feeding between days 1 and 3. This technique avoids the need for repeated sedation and general anaesthesia; it also negates the long waiting time for defect closure whilst avoiding the possible complications associated with the use of staged surgical closure.

## Data Availability

Raw data is available on request.
